# A case of right hepatic duct entering cystic duct successfully treated by laparoscopic subtotal cholecystectomy through preoperatively placed biliary stent

**DOI:** 10.1186/s40792-020-00994-8

**Published:** 2020-09-25

**Authors:** Hiroki Hirao, HiroHisa Okabe, Daisuke Ogawa, Daisuke Kuroda, Katsunobu Taki, Shinjiro Tomiyasu, Masahiko Hirota, Taizo Hibi, Hideo Baba, Hiroki Sugita

**Affiliations:** 1 Department of Surgery, Kumamoto Regional Medical Center, 5-16-10 Honjo, Chuo-Ku, Kumamoto, 860-0811 Japan; 2 Department of Pediatric Surgery and Transplantation, Kumamoto University Graduate School of Medical Sciences, 1-1-1 Honjo, Chuo-ku, Kumamoto, 860-8556 Japan; 3 Department of Gastroenterological Surgery, Graduate School of Medical Sciences, Kumamoto University, 1-1-1 Honjo, Kumamoto, 860-8556 Japan

**Keywords:** Cystic duct anomaly, Right hepatic duct, Laparoscopic cholecystectomy, Endoscopic nasobiliary drainage catheter

## Abstract

**Background:**

Laparoscopic cholecystectomy is a well-established surgical procedure and is one of the most commonly performed gastroenterological surgeries. Therefore, strategy for the management of rare anomalous cystic ducts should be determined.

**Case presentation:**

A 56-year-old woman was admitted to our hospital owing to upper abdominal pain and diagnosed with acute cholecystitis. Magnetic resonance cholangiopancreatography suspected that several small stones in gallbladder and the right hepatic duct drained into the cystic duct. Endoscopic retrograde cholangiopancreatography confirmed the cystic duct anomaly, and an endoscopic nasobiliary drainage catheter (ENBD) was placed at the right hepatic duct preoperatively. Intraoperative cholangiography with ENBD confirmed the place of division in the gallbladder, and laparoscopic subtotal cholecystectomy was safely performed.

**Conclusions:**

The present case exhibited rare right hepatic duct anomaly draining into the cystic duct, which might have caused biliary tract disorientation and bile duct injury (BDI) intraoperatively. Any surgical technique without awareness of this anomaly preoperatively might insufficiently prevent BDI, and preoperative ENBD would facilitate safe and successful surgery.

## Background

Variations of the bile duct before hepatobiliary surgery should be recognized before the hepatobiliary surgery to prevent postoperative complications. A rare cystic duct anomaly increases the risk of bile duct injury (BDI) [1]. However, congenital anomaly of the right hepatic duct (RHD) entering the cystic duct is very rare. Only 10 patients have undergone laparoscopic cholecystectomy on variant cystic ducts till date [2]. Here, we report a case of RHD entering cystic duct successfully treated with laparoscopic cholecystectomy using a preoperatively placed endoscopic retrograde biliary drainage tube inside the RHD.

## Case presentation

**Fig. 1:**
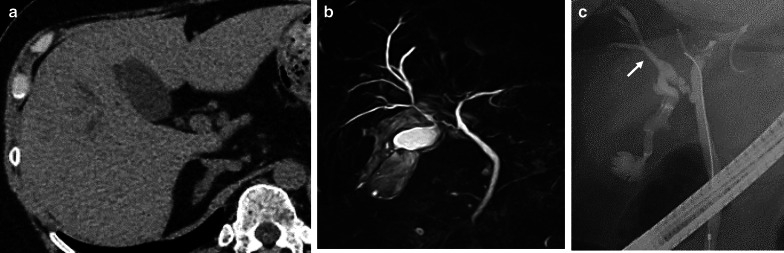
Preoperative imaging of the anomalous cystic duct. **a** Plain abdominal computed-tomography revealing a dilated right hepatic duct. **b **Magnetic resonance cholangiopancreatography showing the congenital anomaly of the right hepatic duct entering the cystic duct. **c** Endoscopic retrograde cholangiopancreatography confirmed the right hepatic duct entering the cystic duct. An arrow indicates right hepatic duct

**Fig. 2:**
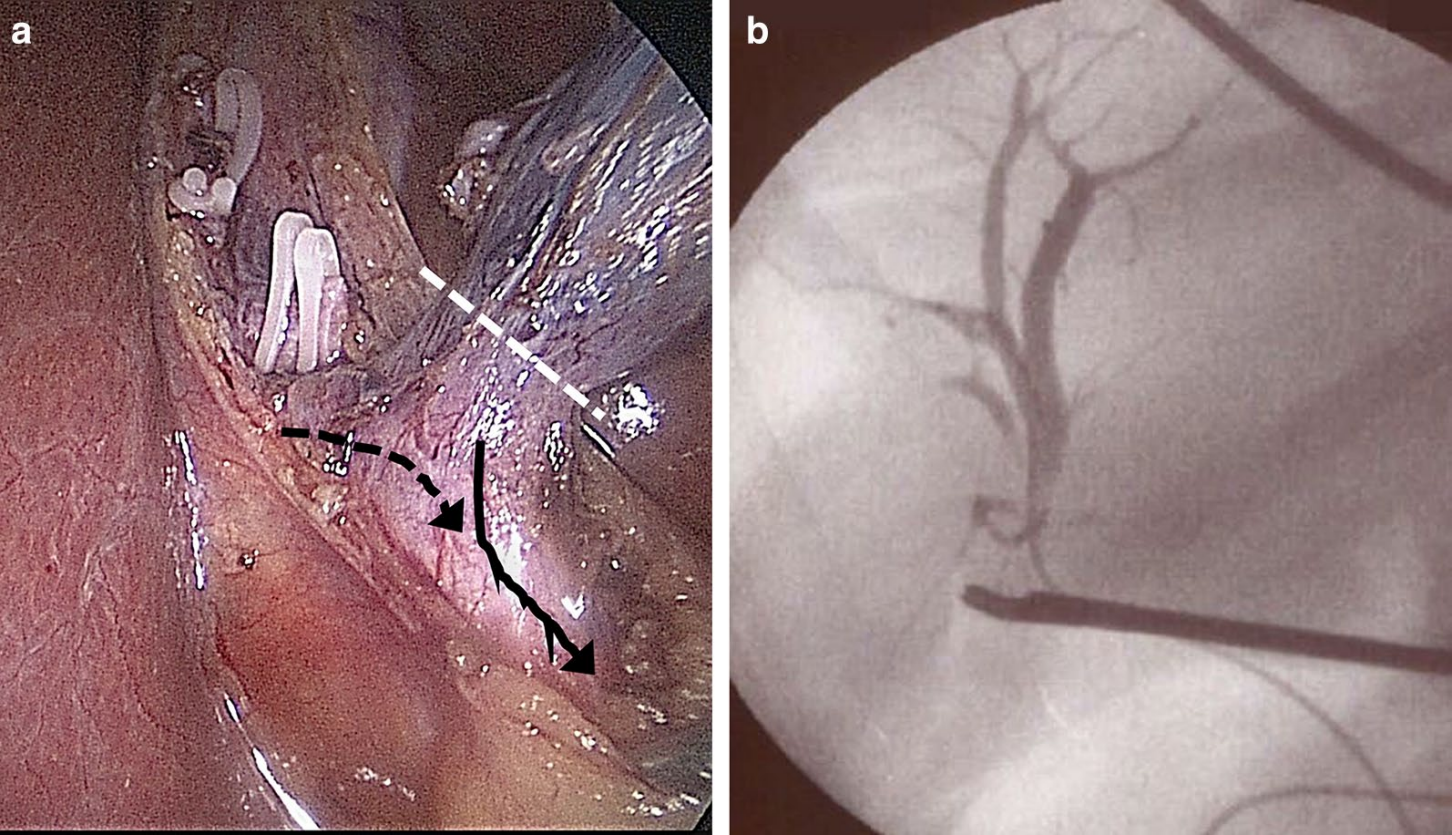
Intraoperative findings of laparoscopic cholecystectomy. **a** An image showing the confluence of the right hepatic duct on the cystic duct. The white dotted line indicates the cut line of the gall bladder for subtotal cholecystectomy. The black arrow indicates the cystic duct. The black dotted arrow indicates the right hepatic duct entering the cystic duct. **b** Intraoperative cholangiography using an ENBD catheter confirming that the forceps grasping the intended cut line of the gallbladder was distant from the ENBD catheter

**Fig. 3:**
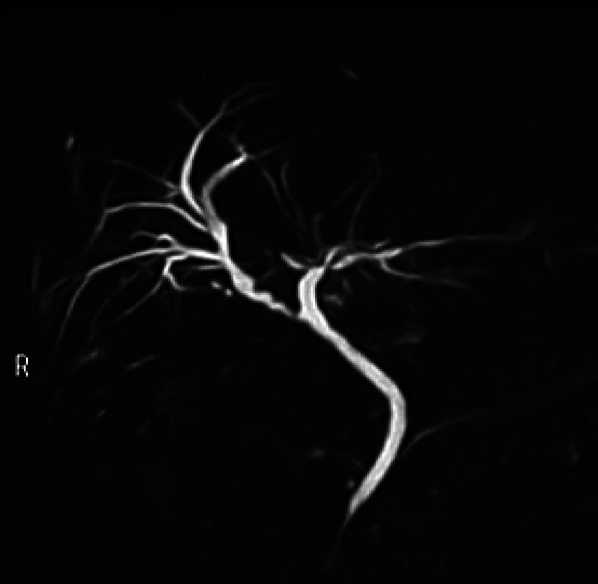
Postoperative imaging of the biliary tract. A representative image of MRCP performed 9 months postoperatively showing the remnant cystic duct functioning as the right hepatic duct

A 56-year-old woman was admitted to our hospital owing to upper abdominal pain. She did not have fever. Her Murphy’s sign was negative. Laboratory data were as follows: white blood cell count, 72.0 × 10^3^/μL, and C-reactive protein, 0.23 mg/dL. Plain abdominal computed tomography (CT) revealed dilation of both the RHD and gall bladder (Fig. 1a). She was admitted to our hospital for observation. On the next day, contrast-enhanced CT and magnetic resonance cholangiopancreatography (MRCP) were performed, with the latter detecting a congenital anomaly of the RHD entering into the cystic duct (Fig. 1b). Although several small stones in gallbladder was found, no choledocholithiasis was confirmed to cause biliary obstruction. Endoscopic retrograde cholangiopancreatography revealed that the RHD entered the cystic duct. Therefore, an endoscopic nasobiliary drainage (ENBD) tube was placed inside the RHD. Laparoscopic cholecystectomy was planned. The cystic duct was carefully dissected, and RHD bifurcation was confirmed (Fig. 2a). Intraoperative cholangiography was performed using an ENBD catheter, after grabbing the gallbladder neck with forceps at 10 mm away from the confluence. Intraoperative cholangiography revealed that the catheter in the RHD was not grabbed by forceps (Fig. 2b). The Hartmann’s pouch of the gallbladder was clipped and divided, and the ENBD catheter was removed. Laparoscopic subtotal cholecystectomy was safely performed. The patient was discharged without any complication. At 1 month postoperatively, the patient complained abdominal pain and high fever. Imaging analysis suspected biliary obstruction at the cystic duct due to choledocholithiasis which was also suspected at the first visit preoperatively. A plastic biliary stent was placed, resulting in immediate disappearance of symptom. The stent was removed 3 months postoperatively; however, abdominal pain recurred. Imaging analysis revealed that the RHD was dilated and cystic duct stenosis was suspected. The plastic stent was placed again, and further investigations for malignant diseases were performed. No malignant diseases were found. The patient was observed for 3 months, and the stent was removed again. One month after the last stent removal, MRCP revealed no RHD dilatation (Fig. 3). Sustained swelling of biliary tract after surgery might mainly be the cause of stenosis. The patient has been free from any symptom for a year after MRCP.

## Discussion

Laparoscopic cholecystectomy is a well-established surgical procedure, and strategies to prevent BDI have been well-determined [3]. Although the critical view of safety is globally accepted as a standardized concept [4], the current cystic duct anomalies require exceptional strategies to prevent BDIs. In this case, a rare RHD anomaly entering the cystic duct was confirmed preoperatively, and the patient was successfully treated with endoscopic surgery using an ENBD that was preoperatively placed as a biliary guide. CT images during the first presentation showed a dilated RHD and a distended gall bladder. This finding implies the possibility of cystic duct anomalies that combined the right biliary branch draining together into the common hepatic duct (CHD), unless the gallbladder compressed the RHD, which is diagnosed as Mirrizi syndrome.

**Fig. 4:**
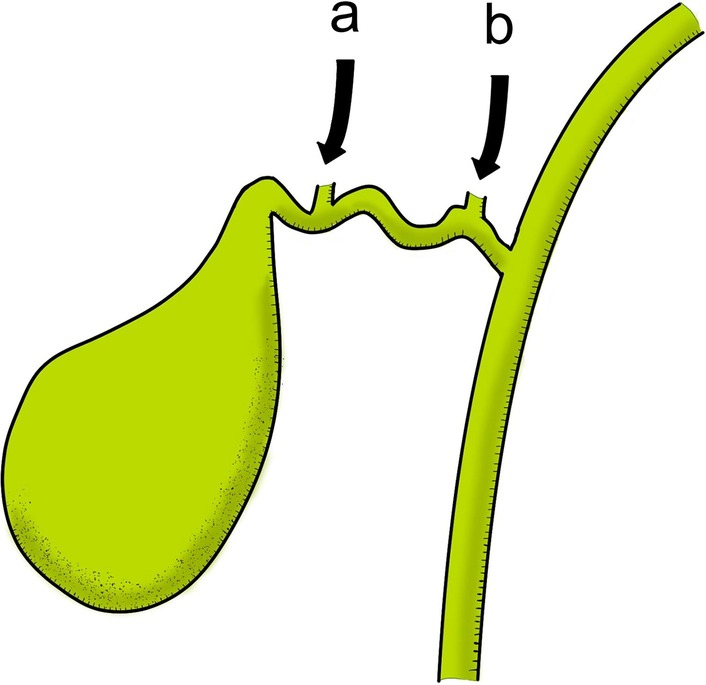
A schema of the confluence pattern of the right hepatic duct on the cystic duct. There were two patterns of the confluence based on the previous four reports. Three out of four (75%) showed the right hepatic duct entering the distal side of the cystic duct (a). One out of four (25%) showed the right hepatic duct entering the proximal side of the cystic duct (b)

A well-known anomalous cystic duct requiring attention to prevent BDI during a laparoscopic cholecystectomy is characterized by the combination of the right posterior segmental duct (RPSD) draining into the CHD and cystic duct entering into RPSD. A previous investigation of cholangiograms on donors revealed that the rarity of RPSD draining directly into CHD was 6%, of which the anomalous posterior branch is close to cystic duct based on the anatomical positioning. Conversely, the rarity of RPSD or RHD entering cystic duct, as in the current case, is 2% or < 1%, respectively [5]. The real percentage of the cystic duct anomaly, as in the current case, is unclear due to its high rarity. Ten cases have been reported till date to be similar to the current case showing RHD entering cystic duct [2]. Intriguingly, one out of four cases (25%) showed RHD entering the proximal side of the cystic duct, causing a wide range of safe cut lengths of the cystic duct, implying that critical complications are less likely to occur intraoperatively. In contrast, three out of four cases (75%) showed RHD entering the distal side of the cystic duct, causing a very short range of safe cut length of the cystic duct, implying that unintended division of the bile flow in RHD is likely to occur intraoperatively (Fig. 4). In such cases, cutting cystic duct purposing to insert a catheter for intraoperative cholangiogram may cause serious complications even under laparotomy, because the cystic duct almost functions as the RHD and cannot be cut. Therefore, these cases, including our case, required subtotal cholecystectomy. Among the ten cases of anomalous RHD on the cystic duct, laparoscopic surgery was successfully performed in four [6–8]. The anomaly was confirmed by cholangiography using the percutaneous transhepatic gallbladder drainage catheter in two cases. Details of the intraoperative cholangiography procedure were not available in the other two reports. Based on these observations, intraoperative cholangiography should be carefully performed depending on the confluence position of the RHD on the cystic duct. We successfully performed laparoscopic cholecystectomy using an ENBD catheter placed during ERCP, which was aimed to diagnose an anomalous cystic duct preoperatively. This strategy decreases stress levels and prevents performance of technically difficult procedures intraoperatively [9].

## Conclusions

The current case indicates that comprehension of cystic duct anomaly is mandatory; the rare anomalous cystic duct, as in the current case, might cause intraoperative disorientation, leading to BDI. This very rare anomaly that allows the RHD to enter the cystic duct should be carefully considered. Although confirming critical view of safety and performing bailout procedure might be enough for the current case, it is possible that an experienced surgical technique and knowledge of various possible anomalies of cystic duct could have been required, if cholecystitis was more challenging at surgery. If the careful investigation was required for a case suspected to have very rare anomaly of cystic duct, ERCP is performed to confirm the anatomy of biliary tract. It is not difficult to place the ENBD for such case. We suggest that preoperative placement of ENBD is a safe and certain strategy to make sure the biliary system anatomy during laparoscopic cholecystectomy.

## Data Availability

All data and materials are available.

## Abbreviations

ENBD: Endoscopic nasobiliary drainage catheter
BDI: Bile duct injury
RHD: Right hepatic duct
CT: Computed tomography
MRCP: Magnetic resonance cholangiopancreatography
CHD: Common hepatic duct
